# Permutation-Invariant Cascaded Attentional Set Operator for Computational Nephropathology

**DOI:** 10.34067/KID.0000000668

**Published:** 2025-03-03

**Authors:** Samira Zare, Huy Q. Vo, Nicola Altini, Vitoantonio Bevilacqua, Michele Rossini, Francesco Pesce, Loreto Gesualdo, Sándor Turkevi-Nagy, Jan Ulrich Becker, Chandra Mohan, Hien Van Nguyen

**Affiliations:** 1 Department of Electrical and Computer Engineering, University of Houston, Houston, Texas; 2 Electronic and Information Engineering Department, Polytechnic University of Bari, Bari, Italy; 3 Department of Emergency and Organ Transplantation, University of Bari Aldo Moro, Bari, Italy; 4 Division of Renal Medicine, Fatebenefratelli Isola Tiberina – Gemelli Isola, Rome, Italy; 5 Department of Pathology, University of Szeged, Szeged, Hungary; 6 Institute of Pathology, University Hospital of Cologne, Cologne, Germany; 7 Department of Biomedical Engineering, University of Houston, Houston, Texas

**Keywords:** artificial intelligence

## Abstract

**Key Points:**

- Permutation-invariant cascaded attentional set operator (PICASO) is a versatile set operator that uses Transformers to dynamically aggregate histopathologic features from a set of glomerular crops.
- For detecting active crescent in patients with IgA nephropathy on internal and external validation sets, PICASO achieved an area under the receiver-operating characteristic curve of 0.99 and 0.96, respectively.
- In the case-level classification of antibody-mediated rejection in kidney transplants, PICASO performed well, with an area under the receiver-operating characteristic curves of 0.97.

**Background:**

The advent of digital nephropathology offers the potential to integrate deep learning algorithms into the diagnostic workflow. We introduce permutation-invariant cascaded attentional set operator (PICASO), a novel permutation-invariant set operator to dynamically aggregate histopathologic features from instances. We applied PICASO to two nephropathology scenarios: detecting active crescent lesions in sets of glomerular crops with IgA nephropathy (IgAN) and case-level classification for antibody-mediated rejection (AMR) in kidney transplant.

**Methods:**

PICASO is a Transformer-based set operator that aggregates features from sets of instances to make predictions. It uses initial histopathologic vectors as a static memory component and continuously updates them on the basis of input embeddings. For active crescent detection in patients with IgAN, we obtained 6206 periodic acid–Schiff–stained glomerular crops (5792 no active crescent, 414 active crescent) from three different health institutes. For the AMR classification, we have 1655 periodic acid–Schiff–stained glomerular crops (769 AMR and 886 non-AMR images) from 89 biopsies. The performance of PICASO as a set operator was compared with other set operators, such as DeepSet, Set Transformer, DeepSet++, and Set Transformer++, using metrics including area under the receiver-operating characteristic curve (AUROC), area under the precision-recall curves, recall, and accuracy.

**Results:**

PICASO achieved superior performance in detecting active crescent in patients with IgAN, with an AUROC of 0.99 (95% confidence interval [CI], 0.98 to 0.99) on internal validation and 0.96 (95% CI, 0.95 to 0.98) on external validation, significantly outperforming other set operators (*P* < 0.001). It also attained the highest AUROC of 0.97 (95% CI, 0.90 to 1.0, *P* = 0.02) for case-level AMR classification. The area under the precision-recall curve, recall, and accuracy scores were also higher when using PICASO, and it significantly outperformed baselines (*P* < 0.001).

**Conclusions:**

PICASO can potentially advance nephropathology by improving performance through dynamic feature aggregation.

## Visual Abstract

**Figure:**
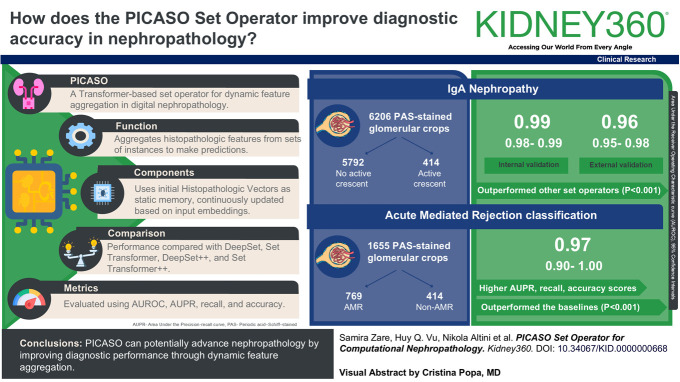

## Introduction

Nephropathology involves extracting diagnostic, prognostic, and theranostic information from diverse datasets, including paraffin-embedded tissue sections. The advent of bright-field histology scanners for paraffin-embedded images has facilitated the use of computer vision techniques, offering significant advantages, such as rapid analysis of large datasets. Owing to the large dimensions of the whole slide images (WSIs) obtained from biopsies (*e.g*., 100,000×100,000 pixels), these images are typically divided into smaller tiles for more effective analysis.^1–5^ In this regard, processing variably sized, often large, sets of tiles from a single WSI is common in various tasks, such as the Banff Classification of Allograft Pathology,^6,7^ the Oxford Classification of IgA nephropathy (IgAN),^8^ the International Society of Nephrology/Renal Pathology Society classification of lupus nephritis,^9^ or the RPS classification of diabetic nephropathy.^10^ Analyzing these sets requires novel architectures capable of handling varying numbers of instances while being permutation invariant, meaning the order of instances is irrelevant to the analysis.^1,11–14^ The use of permutation-invariant operators (or set operators) in nephropathology is well established.^1,14–17^ Specifically, these approaches involve an aggregation operator that is invariant to instance permutation. For example, Bunescu and Mooney^18^ used simple pooling operators, such as max or its variant, top-k max, to identify specific instances to form the prediction. Similarly, other operators, such as mean, log-sum-exp, and noisy logical operators,^19,20^ were also used to merge the representations of all instances into a single WSI representation. Ilse *et al.*^13^ introduced AttentionMIL, which incorporates attention pooling operators to compute weighted sums of each instance’s representation, with weights learned from the instance representations using a deep learning model. Subsequent studies, such as clustering-constrained attention multiple instance learning,^1^ and more recent Transformer-based techniques^15–17^ also leverage attention mechanisms, further supporting the potential of attention-based aggregation modules to achieve permutation invariance.

Previous set operators^11,12,21^ have several limitations. They could not dynamically adapt their architecture once trained, resulting in poor generalization during inference, especially on external validations. This might cause challenges for tasks that involve learning the necessary knowledge for a task and then adapting decisions on the basis of the morphological variations in each case. Furthermore, the Transformer architecture^22^ is not inherently permutation invariant, making them unsuitable for set-input tasks without modifications.^12,21^ Motivated by these limitations, we developed a novel Transformer-based architecture, called permutation-invariant cascaded attentional set operator (PICASO). This architecture can dynamically aggregate histopathologic features from sets of instances. It leverages histopathologic vectors (HiVe) as the query^12,22^ to achieve permutation invariance and reduce the computational complexity. PICASO dynamically updates the HiVe by comparing it with embeddings of input instances. Intuitively, this dynamic adaptation mirrors the way nephropathologists adjust their assessments when confronted with specific characteristics of a set of instances, such as staining differences or morphological variations.

PICASO is a versatile aggregation module that can be used for a wide range of nephropathology tasks. We validated its performance in two tasks: detecting active crescents in IgAN biopsies^8^ and diagnosing antibody-mediated rejection (AMR).^6,7^ Previous works on IgAN lesion detection have underscored the potential of deep learning for developing automated scoring systems^5^ and integrating these models to assist pathologists in their daily workflows.^23^ However, these studies primarily focused on individual instances rather than set-based approaches. Our results demonstrate an improvement compared with classifying each instance individually.^24^ Our formulation draws inspiration from anomaly detection systems capable of identifying glomerular crops with specific lesions. Regarding the AMR classification task, earlier studies^16^ evaluated performance with other set pooling operators. By contrast, we show that PICASO can enhance performance. The dynamic HiVe resulted in improved generalization and more robust performance during inference. This makes PICASO a promising architecture for a wide range of clinical and research applications in nephropathology.

## Methods

### PICASO Architecture Development

**Figure 1:**
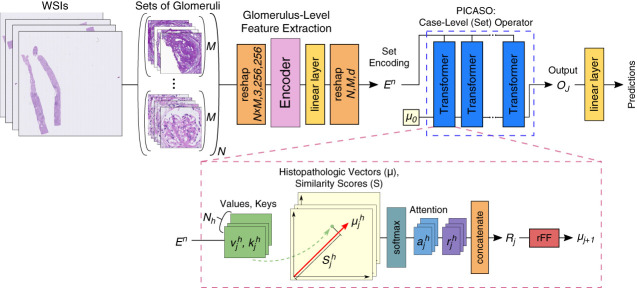
**Overview of the overall deep learning model architecture.** We began by sampling M images across N cases and encoded each image as an individual batch element. After passing our encoded images through a linear layer, we concatenated the M encoded image embeddings to form N sets. We then passed the sets through PICASO, which consisted of a cascade of multihead attention Transformers. PICASO started with initial static HiVe $\mu_{0}$, which was learned during training. At each step, a new HiVe was computed on the basis of the input sets and used in the subsequent Transformer. After J steps of updating the HiVe in PICASO, the output was passed through a linear layer to generate the final prediction for each set. This aggregation process in PICASO aimed to replicate a nephropathologist’s cognitive workflow, where they first learn initial rules for a task and subsequently adapt their decisions on the basis of the morphological variations of each image. To compare the performance of PICASO with other set operators, the PICASO block was replaced with each baseline operator. More details about the architecture are present in the Supplemental Methods. HiVe, histopathologic vectors; PICASO, permutation-invariant cascaded attentional set operator; WSIs, whole slide images.

Our architecture is designed to process cases with varying numbers of instances, ensuring that the order of instances does not affect the output. Figure 1 illustrates how PICASO can be used as a set operator atop encoders like EfficientNet to aggregate embeddings from each set. Let $E^{n}\in R^{M\times d_{E}}$ represent embeddings for a set of M instances from the $n^{th}$ case. The value of M can vary across different cases, and the Transformer architecture can accommodate this variability. These embeddings are input into PICASO, which uses HiVe to aggregate information from each set into a fixed length ($N_{HV}$). As shown in Figure 1, PICASO comprises J Transformer blocks,^22^ where HiVe $\mu_{j}\in R^{N_{HV}\times d_{\mu_{j}}}$ is used as the query and embeddings $E^{n}$ serve as key and value matrices. Each Transformer block uses a multihead attention mechanism to update the HiVe from the previous block:

$$\mu_{j+1}=\text{Transformer}\left(\mu_{j},E^{n},E^{n}\right)\;j=0,\ldots ,J$$

with j indicating the step. HiVe contains discriminative features for histopathological key concepts. The initial HiVe, denoted as $\mu_{0}$, is parametrized and initialized using the Kaiming initialization method.^25^ We aim to learn the initial HiVe during training. The number of steps J is a hyperparameter that can be empirically determined on the basis of model performance. Finally, we pass the aggregated features through a linear layer to generate predictions. More technical details about the PICASO architecture is provided in the Supplemental Methods.

Initial HiVe can be thought of as a static memory component that encapsulates the histopathology characteristics of a specific task. It mimics how pathologists learn the fundamental features and tissue morphology for each task. The dynamic aspect comes into play as we continuously update the HiVe on the basis of the input embeddings. Intuitively, this dynamic adaptation mirrors the way nephropathologists evaluate each image, considering the key attributes while accounting for the unique aspects influenced by variables such as staining methods or the scanner used. In essence, our goal is to replicate the cognitive workflow of expert nephropathologists.

In addition, using HiVe as a query in Transformer allows to retain linear time complexity $O\left(M\times N_{HV}\right)$, where $N_{HV}<M$. In the Supplemental Materials (Properties of PICASO), we prove that PICASO is a valid set operator, *i.e*., it can handle sets with varying numbers of instances and is permutation invariant (property 1). We also show that PICASO can be described as a generalization of K-means clustering (property 2), confirming the adaptability of PICASO to variations in histopathological images. Results of a synthetic clustering experiment are shown in Supplemental Figure 1.

### Dataset Generation and Annotation

#### IgAN

This study included 102 renal biopsies diagnosed with primary IgAN according to the Oxford Classification.^8^ These biopsies were collected from the archives of the Institute of Pathology in Cologne (Germany), the Institute of Pathology in Szeged (Hungary), and the Nephrology Department in Bari (Italy). We focused exclusively on periodic acid–Schiff (PAS)^26^ staining because the Oxford score is, by definition, restricted to PAS. PAS sections from all biopsies were scanned with either a NanoZoomer Scanner (Hamamatsu, Herrsching am Ammersee, Germany) for Cologne and Bari biopsies or a Pannoramic Midi Slide Scanner (3DHISTECH, Budapest, Hungary) for Szeged biopsies, yielding a resolution of approximately 0.25 μm per pixel. All biopsies met the minimum criteria for Oxford scoring, containing at least eight glomeruli. These 102 biopsies corresponded to a total of 308 WSIs. Table 1 provides a summary of these three cohorts. An expert nephropathologist (J.U. Becker) annotated all biopsies for glomeruli using QuPath^27^ and a Cintiq Pro 16-inch pen tablet (Wacom, Düsseldorf, Germany). The annotations included sclerosed and nonsclerosed glomeruli, excluding empty Bowman capsules without a discernible tuft element. All glomerular crops were then labeled by the expert nephropathologist on the Labelbox platform (www.labelbox.com, last accessed January 27, 2024). In total, 6206 glomerular crops were expert annotated for mesangioproliferation (M), endocapillary hypercellularity (E), segmental glomerulosclerosis (S), and active crescent (C). Further details are given in a previous publication.^24^

**Table 1:** Summary of the IgA nephropathy dataset

Cohort	Biopsies	Glomeruli	M-Lesion			E-Lesion		S-Lesion			C-Lesion	
			Null	noM	yesM	noE	yesE	GGS	noGS	SGS	noC	yesC
Bari	7	365	90	241	34	360	5	40	307	18	336	29
Cologne	275	4525	1233	1922	1370	4417	108	1000	2969	556	4250	275
Szeged	26	1316	537	329	450	1242	74	491	662	163	1206	110
Total	308	6206	1860	2492	1854	6019	187	1531	3938	737	5792	414

The biopsy cohorts were collected from three health institutions: Bari (Italy), Cologne (Germany), and Szeged (Hungary), to cover different stain appearances. Biopsies were all periodic acid–Schiff stained, but they were scanned using different scanners at each institution. Our experiments focus on detecting active crescent (C-lesion). C, crescent; E, endocapillary hypercellularity; GGS, global glomerulosclerosis; M, mesangial hypercellularity; GS, glomerulosclerosis; SGS, segmental glomerulosclerosis.

#### AMR

We used a set of glomerular crops obtained from patients receiving kidney transplant for which we knew the case-level ground truth labels. This ground truth label was determined by the Banff Classification in its 2018 iteration.^7^ Our dataset was obtained from stained kidney transplant biopsies from the archives of the Institute of Pathology in Cologne. It comprised a total of 89 biopsies, of which 51 had chronic active, chronic, or active AMR, while the remaining 38 were labeled non-AMR. All biopsies met the minimum sample criteria as defined by the Banff Classification, *i.e*., having at least seven glomeruli and at least one artery.^7^ All biopsy sections were consistently cut to a thickness of 2 μm and were PAS stained in the same pathology laboratory over a 2-year period. Micrographs were captured from all nonglobally sclerosed glomeruli, which were at least four levels apart, at a resolution of 1024×768 pixels. This resulted in a total of 1655 glomerular crops (769 AMR and 886 non-AMR images).

### Experimental Design

#### Training and Validation Sets

**Figure 2:**
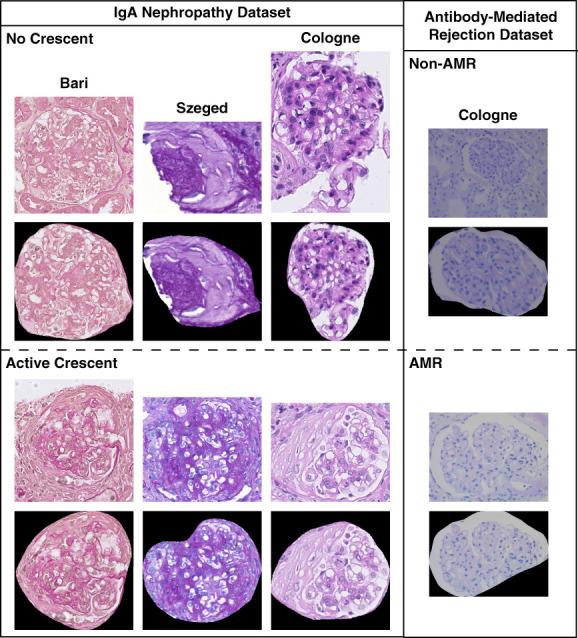
**Examples of glomerular crops for the IgAN dataset and AMR dataset.** The IgAN dataset was collected from three health institutions, Bari (Italy), Cologne (Germany), and Szeged (Hungary), to cover different stain appearances. Our experiments focused particularly on detecting active crescent (C-lesion) in patients with IgAN. The AMR dataset was collected from the Institute of Pathology in Cologne and used for a binary classification task (non-AMR versus active, chronic active, and chronic AMR). All biopsies were PAS stained, but they were scanned using different scanners at each institution. The second rows display the masked images, where pixels outside the glomerular area were removed. AMR, antibody-mediated rejection; IgAN, IgA nephropathy; PAS, periodic acid–Schiff.

We partitioned the glomerular images into five folds for cross-validation, ensuring that crops from the same WSI were not shared between training and validation (approximately 80% training and approximately 20% validation in each fold). All images were manually masked to remove pixels outside the glomerular area (Figure 2). We applied zero padding to render the glomerular crop into a square shape. The images were resized to 256×256 pixels with no stretch. In our experiments, we first create sets of glomerular crops for each case as inputs to our model. We pursued a Monte Carlo sampling scheme and randomly sampled M unique images from a given WSI at each training iteration. The value of M varied at each iteration to ensure the model encountered different image combinations and could handle variability in the number of instances. If there were fewer than M instances, we used online augmentation (*e.g*., random resized crop, random vertical flip, and random rotation) to create additional images.

For the IgAN lesion detection, we created sets using M∈[3,7] images without active crescent and one image with active crescent. In total, it made a set of M∈[4,8] images for each case. The selected images may contain none or any of the other Oxford Classification lesions (M, E, or S). We did not have any control over these other lesions for creating sets, making our experiment more realistic and challenging. The ground truth label for each set would be a one hot vector indicating which glomerular image contains active crescent. We created 500 sets from the Bari dataset, 1000 sets from the Szeged dataset, and 2500 sets from the Cologne dataset for training (total N=4000 sets). Similarly, for each validation fold, we created 160 sets from Bari, 300 sets from Szeged, and 800 from Cologne for test (total N=1260). In a separate experiment, we used the Bari dataset as an external validation dataset because it had a smaller size.

Similarly, for the AMR classification, we created sets of M∈[4,8] randomly selected glomerular images for each case. The ground truth case-level label for each set would be either 0 (non-AMR) or 1 (active, chronic active, and chronic AMR). We created N=2500 sets for training and N=500 sets for testing.

#### Deep Learning Model Design

During training, a batch size of 32 cases was used. We used the EfficientNetv2-rw-s encoder for the IgAN dataset and EfficientNet-B3 encoder for the AMR dataset. Both encoders were sourced from PyTorch ImageNet pretrained models^28^ and fine-tuned end to end using a binary cross entropy loss function with Adam optimizer for 20 epochs with a learning rate of $1\times 10^{-4}$. We used d=512 for IgAN and d=256 for AMR as the hidden dimensions. We had $N_{h}=8$ attention heads in Transformers. To prevent overparameterization, the parameters were shared across the Transformers in PICASO. The same architecture designs were used for baseline set operators^11,12,21^ to ensure a fair comparison. DeepSet^11^ and DeepSet++^21^ use max, mean, or sum operations for aggregation, whereas Set Transformer^12^ and Set Transformer++^21^ are attention based.

The number of HiVe ($N_{HV}$) determines the output dimensionality, which should be compatible with the target label for each task. For IgAN, we used $N_{HV}=1$. Because the final HiVe represents the weighted mean of the glomerular images for each case (Supplemental Materials), we computed the L1 distance between each image embedding and this mean to detect images with C-lesion. The deviations were then passed through the output layer. For the AMR classification, we used $N_{HV}=1$, which made the network output aligned with ground truth labels. Learning curves are provided in Supplemental Figure 2. We used Python 3.8 and PyTorch 1.14.0 library. The source code is freely accessible at https://github.com/hula-ai/PICASO.

### Model Evaluation

For IgA lesion detection, the performances of the models were evaluated using three criteria: area under the receiver-operating characteristic curve (AUROC), area under the precision-recall (AUPR) curve, and recall. For the case-level AMR classification, we used AUROC, AUPR curve, and accuracy to measure the performances. All *P* values were two sided, and values <0.05 were considered significant.

## Results

### IgAN Active Crescent Detection

First, we used datasets from three institutes for both training and validation (no external validation). The AUROC for detecting C-lesion was highest when using PICASO as the set operator (0.99; 95% confidence interval [CI], 0.98 to 0.99), followed by DeepSet’max_w_ (0.96; 95% CI, 0.94 to 0.98), DeepSet’mean_w_ (0.96; 95% CI, 0.94 to 0.98), DeepSet++’max (0.96; 95% CI, 0.94 to 0.98), Set Transformer++ (0.96; 95% CI, 0.94 to 0.98), and Set Transformer (0.94; 95% CI, 0.92 to 0.96). The AUPR curve was 0.94 (95% CI, 0.93 to 0.95) for PICASO, 0.90 for Set Transformer++ (95% CI, 0.84 to 0.96), 0.88 for DeepSet++’max (95% CI, 0.84 to 0.92), 0.88 for DeepSet’mean (95% CI, 0.82 to 0.94), and 0.84 for Set Transformer (95% CI, 0.74 to 0.94). PICASO also achieved a high recall (0.87; 95% CI, 0.86 to 0.88), followed by DeepSet++’sum (0.87; 95% CI, 0.81 to 0.93), DeepSet’mean (0.82; 95% CI, 0.74 to 0.90), Set Transformer++ (0.80; 95% CI, 0.74 to 0.86), and Set Transformer (0.79; 95% CI, 0.71 to 0.87). More detailed results are reported in Table 2. All *P* values were <0.001. Breakdown of the performance in detecting C-lesion for each institute are listed in Supplemental Table 1. We note that a convolutional neural network model (with the same encoder) for classifying these glomerular crops individually for active crescent could achieve an AUROC of 0.92 and AUPR curve of 0.76.^24^

**Table 2:** Results for detecting active crescent (C-lesion)

Model	No. of Parameters	No External Validation			External Validation (Bari)		
		AUPR Curve	AUROC	Recall	AUPR Curve	AUROC	Recall
DeepSet’max_w_^11^	24.9M	0.8740±0.05	0.9635±0.01	0.8115±0.05	0.8239±0.04	0.9471±0.01	0.7630±0.04
DeepSet’max^11^	24.1M	0.8441±0.05	0.9522±0.01	0.7844±0.05	0.8139±0.05	0.9289±0.01	0.7580±0.04
DeepSet’mean_w_^11^	24.9M	0.8753±0.03	0.9557±0.01	0.8052±0.03	0.6504±0.02	0.8948±0.01	0.6210±0.02
DeepSet’mean^11^	24.1M	0.8755±0.03	0.9547±0.01	0.8166±0.04	0.8010±0.05	0.9413±0.01	0.7420±0.03
DeepSet++’max^21^	24.6M	0.8847±0.02	0.9611±0.01	0.7849±0.03	0.8350±0.04	0.9181±0.04	0.7484±0.01
DeepSet++’sum^21^	24.6M	0.7217±0.06	0.9146±0.03	0.8739±0.03^a^	0.6489±0.03	0.8884±0.01	0.8280±0.02
Set Transformer^12^	24.1M	0.8387±0.05	0.9415±0.01	0.7866±0.04	0.7003±0.09	0.9253±0.01	0.7200±0.06
Set Transformer++^21^	28.4M	0.9000±0.03	0.9606±0.01	0.7995±0.03	0.7818±0.03	0.8898±0.01	0.6704±0.01
PICASO	24.6M	0.9367±0.01^a^	0.9860±0.01^a^	0.8685±0.01	0.8718±0.02^a^	0.9594±0.01^a^	0.8619±0.02^a^

In the experiment without external validation, images from all three institutes were used for training and validation. For the experiment with external validation, the smaller (Bari) dataset was held out for validation. PICASO outperformed in both experiments, especially in the setting with external validation. This suggests that the ability of PICASO to update histopathologic vectors on the basis of the input helped the model adapt to unseen input variations, such as differences in color appearance in the glomerulus images. The number of trainable parameters for PICASO is comparable with other models, and it did not increase the model size. DeepSet’ (∙) and DeepSet’++ (∙) refer to the aggregation mechanisms used in the model, with DeepSet’ (∙)_w_ indicating the use of a weighted pooling function. AUPR, area under the precision-recall; AUROC, area under the receiver operating characteristic curve; PICASO, permutation-invariant cascaded attentional set operator.

a Best performing model.

In the second experiment, we used the Bari dataset as an external validation to evaluate the generalizability. In this experiment, PICASO achieved an AUROC of 0.96 (95% CI, 0.95 to 0.98), outperforming DeepSet’max_w_ (0.95; 95% CI, 0.93 to 0.97), Set Transformer (0.93; 95% CI, 0.91 to 0.95), DeepSet++’max (0.92; 95% CI, 0.84 to 0.98), and Set Transformer++ (0.89; 95% CI, 0.87 to 0.91). In addition, the AUPR curve and recall were 0.87 (95% CI, 0.83 to 0.91) and 0.86 (95% CI, 0.81 to 0.89), respectively, for PICASO. However, the AUPR curve and recall were 0.84 (95% CI, 0.76 to 0.92) and 0.75 (95% CI, 0.73 to 0.75) for DeepSet++’max, 0.82 (95% CI, 0.74 to 0.90) and 0.76 (95% CI, 0.68 to 0.84) for DeepSet’max_w_, 0.78 (95% CI, 0.72 to 0.86) and 0.67 (95% CI, 0.65 to 0.69) for Set Transformer++, and 0.7 (95% CI, 0.52 to 0.88) and 0.72 (95% CI, 0.61 to 0.84) for Set Transformer, respectively (Table 2).

### Case-Level AMR Diagnosis

PICASO demonstrated a remarkable accuracy of 94.52% (95% CI, 88.42% to 100%, *P* < 0.001), followed by DeepSet’max (91.81%; 95% CI, 85.71% to 97.91%), Set Transformer (86.26%; 95% CI, 79.48% to 93.04%), DeepSet++’sum (85.54%; 95% CI, 75.58% to 95.50%), and Set Transformer++ (82.14%; 95% CI, 76.28% to 87.98%). The accuracy metric might not show the complete picture of how different methods perform, especially in the case of imbalanced classes. PICASO achieved an AUROC of 0.97 (95% CI, 0.90 to 1.0; *P* = 0.02), which is better than DeepSet (0.96; 95% CI, 0.95 to 0.97), DeepSet++ (0.89; 95% CI, 0.73 to 0.99), Set Transformer (0.89; 95% CI, 0.81 to 0.97), and Set Transformer++ (0.84; 95% CI, 0.74 to 0.93). PICASO also achieved the highest AUPR (0.97; 95% CI, 0.89 to 1.0; *P* < 0.001), followed by DeepSet (0.96; 95% CI, 0.90 to 1.0), DeepSet++ (0.93; 95% CI, 0.87 to 0.99), Set Transformer++ (0.89; 95% CI, 0.81 to 0.97), and Set Transformer (0.86; 95% CI, 0.78 to 0.94). The results are presented in Table 3. Confusion matrix is also provided in Supplemental Figure 3.

**Table 3:** Results for antibody-mediated rejection case-level classification

Model	No. of Parameters	AUPR	AUROC	Accuracy
DeepSet’max_w_^11^	17.0M	0.9644±0.03	0.9643±0.05	90.35±5.40%
DeepSet’max^11^	16.2M	0.9399±0.03	0.9605±0.03	91.81±3.05%
DeepSet’mean_w_^11^	17.0M	0.9268±0.05	0.9620±0.02	89.83±5.68%
DeepSet’mean^11^	16.2M	0.9297±0.01	0.9472±0.03	89.04±0.29%
DeepSet++’max^21^	19.9M	0.9300±0.03	0.8888±0.03	83.50±6.07%
DeepSet++’sum^21^	19.9M	0.9007±0.09	0.8926±0.08	85.54±4.98%
Set Transformer^12^	16.2M	0.8582±0.04	0.8908±0.04	86.26±3.39%
Set Transformer++^21^	18.3M	0.8917±0.04	0.8383±0.05	82.14±2.93%
PICASO	16.2M	0.9653±0.04^a^	0.9732±0.03^a^	94.52±0.15%^a^

PICASO outperformed the baselines with high accuracy in case-level binary classification of antibody-mediated rejection (non–antibody-mediated rejection versus active, chronic active, and chronic antibody-mediated rejection). The area under the precision-recall curve and area under the receiver operating characteristic curve were also marginally higher than other set operators. The number of trainable parameters for PICASO is comparable with other models, and it did not increase the model size. AUPR, area under the precision-recall; AUROC, area under the receiver operating characteristic curve; PICASO, permutation-invariant cascaded attentional set operator.

a Best performing model.

**Figure 3:**
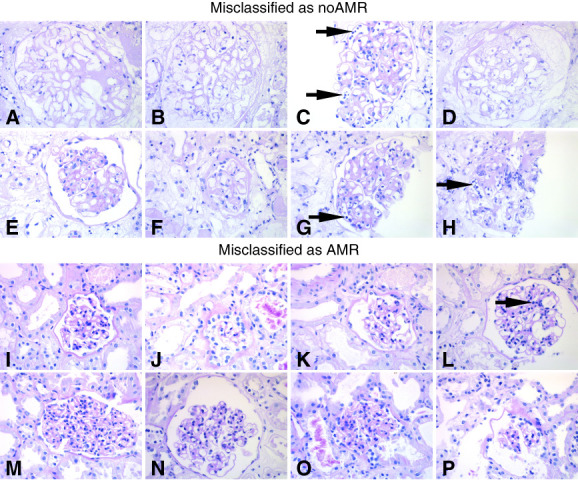
**Examples of misclassified cases in the AMR classification task.** (A–H) Eight glomeruli misclassified as non-AMR (false negative), (I–P) eight glomeruli misclassified as AMR (false positive). For a human nephropathologist, the first case could be clearly identified as AMR on the basis of the accumulation of mononuclear cells in the glomerular capillaries (arrows in C, G, and H); it can be speculated that the model missed this because of the lower cell content (discernible by the missing dense-blue nuclei) in these partially infarcted glomeruli. In the second biopsy, the reason is not readily apparent but could be related to the segmentally high cell mesangial density (arrow in L) in these slightly collapsed glomeruli.

We also asked the expert nephropathologist (J.U. Becker) to evaluate examples of false-positive and false-negative predictions when using PICASO as the set operator, as shown in Figure 3. Arrows were used to point to the potential factors in the glomerular images that contributed to the misclassification.

### Number of Updating Steps in PICASO

**Figure 4:**
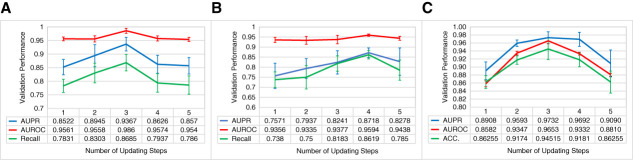
**Variation of performance metrics by changing the number of updating steps (*i.e*., number of Transformers)** J **in PICASO.** Performance did not consistently improve by increasing the number of steps, possibly because of overfitting to set embeddings or deviation of HiVe from true representations. In each experiment, the number of steps with the highest performance was used in PICASO. (A) IgAN C-lesion detection with no external validation data. (B) IgAN C-lesion detection with external validation data. (C) Case-level classification of AMR. Accuracy (ACC.) is divided by 100 for presentation purposes. AUPR, area under the precision-recall; AUROC, area under the receiver operating characteristic curve.

We explored the effect of different numbers of updating steps J in Figure 4. Number of updating steps in PICASO is a hyperparameter that refers to the number of Transformers used to update the initial HiVe. We incrementally increased the number of Transformers until we observed no further improvement. For IgAN C-lesion detection, without and with external validation, three and four steps yielded the best performance. For the AMR classification, PICASO required three steps.

### Number of Images for Each Case

**Figure 5:**
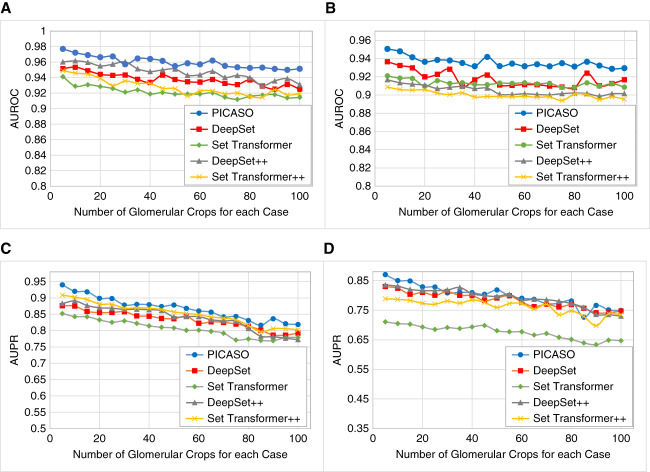
**Variation of AUROC and AUPR curve for different set operators when the number of images for each case was changing.** All models demonstrated robustness against variation in the set size, with only a slight performance drop. All models were trained on sets with a maximum size of eight glomerular images. (A) AUROC for IgAN C-lesion (active crescent) detection without external validation. (B) AUROC for IgAN C-lesion (active crescent) detection with external validation. (C) AUPR curve for IgAN C-lesion (active crescent) detection without external validation. (D) AUPR curve for IgAN C-lesion (active crescent) detection with external validation.

Considering that the number of images per case can vary during inference, we evaluated the robustness of different set operators to accommodate variations, as shown in Figure 5. The results demonstrated that all set operators maintained robustness, with AUROC only about a 0.02–0.03 drop observed for large sets. The AUPR curve showed more variations compared with AUROC.

## Discussion

PICASO is an add-on module that aggregates information from sets of varying lengths into a fixed-length output. It is a versatile operator adaptable to various nephropathology applications where multiple instances need to be analyzed collectively. Previously, set operators were studied^11,12,21^; however, our test results indicated that PICASO outperforms them. The distinct characteristic of PICASO is its dynamic architecture, which can adaptively aggregate histopathologic features on the basis of input image characteristics, similar to how nephropathologists adjust their assessments. Initially, the static HiVe effectively captures histopathological features during training. Through updating steps, it adapts to input variations, such as staining or the scanner used, enabling the network to generalize better to unseen images. Comparing the substantial improvements with the Set Transformer, which is a static Transformer-based module, can highlight the effect of PICASO’s updating steps.

PICASO accurately detected C-lesions in patients with IgAN on both internal and external validation datasets. It also achieved the best performance for case-level classification of AMR, surpassing other set operators.^11,12,21^ These results highlight the effectiveness of PICASO in clinical nephropathology applications. Its performance was also compared with single-glomeruli classification. The findings indicate that analyzing a set of glomerular crops for each case is more accurate than analyzing each crop individually, intuitively similar to how nephropathologists approach the task. Improving the performance in tasks such as C-lesion detection in patients with IgAN or AMR prediction from biopsy samples with less supervision can facilitate timely intervention plans. To the best of our knowledge, there is no set formulation for lesion detection in IgAN and only a few for case-level AMR classification.^16^

The optimal number of updating steps for the best performance is a hyperparameter that requires tuning for each individual task. While these steps enable PICASO to adapt the initial HiVe to the image embeddings, increasing the number of steps did not always lead to improved performance (Figure 4). This plateau or decline in performance could result from the updated HiVe deviating from their appropriate concepts or overfitting to the set embeddings. In addition, PICASO and other set operators^11,12,21^ showed robustness against variations in the number of available images per case. The slight performance drop might be because these models were trained on small sets of up to eight glomerular images. Increasing the set size (compared with the training process) slightly decreased the performance of all models.

Despite the promising results, certain limitations need to be considered. Deep learning models are often black boxes, making it difficult to understand how predictions are made or what features are learned. To evaluate the concepts learned by HiVe, the expert nephropathologist (J.U. Becker) reviewed cases of false-positive and false-negative predictions in AMR classification. In the false-negative case, the lower cell content in the partially infarcted glomeruli was a subtle detail missed by the model. In the false-positive case, high mesangial cell density in slightly collapsed glomeruli likely led to misclassification. These insights suggest that misclassifications could be due to misleading factors in the images rather than fundamental errors in the concept learned by the model. Furthermore, in the Supplemental Methods, we explained that PICASO can be considered a K-means algorithm, making its output interpretable as a weighted mean of instances. In addition, complex deep learning architectures can be expensive to implement. PICASO uses HiVe as a query to reduce the computational complexity and has a linear complexity. The number of model parameters is also similar to that in previous set operators because of weight sharing across the Transformers (Tables 2 and 3). Another limitation is that PICASO was designed for permutation-invariant applications, which means it does not inherently consider spatial correlations between instances.^29^ This could limit performance for tasks requiring spatial awareness, such as diabetic nephropathy analysis. Incorporating positional encoding could address this, although this needs to be validated in future studies.

While we have applied PICASO only to glomerular tasks, it could also be applied to other compartments such as arteries, arterioles, or cortical tubulointerstitium tiles. PICASO’s integration into clinical practice holds the potential to transform nephropathology, particularly in the diagnosis and management of complex kidney diseases. By analyzing sets of glomeruli, as opposed to individual crops, PICASO mirrors the comprehensive approach typically used by nephropathologists, providing a more holistic and consistent assessment of renal pathology. One significant clinical application of PICASO lies in its capacity to standardize diagnostic processes. In daily clinical practice, pathologists often face variations in staining, scanner type, and image quality, which can introduce inconsistencies in interpretation. PICASO’s adaptability to these variables, demonstrated by its updating steps that recalibrate to input variations, ensures more robust performance across different institutions. This could be particularly beneficial in multicenter trials.

This study introduced and evaluated a set operator using our in-house dataset, but further investigations and applications can be defined for future research. Overall, PICASO is a novel approach to computational nephropathology using a Transformer-based architecture. It dynamically aggregates histopathologic features from sets of images by adapting HiVe to the unique characteristics of each case. Our results highlighted PICASO’s versatility and capabilities, representing a significant advancement in digital pathology and offering a more robust, efficient, and adaptable tool for nephropathology analysis.

## Data Availability

Partial restrictions to the data and/or materials apply. Both datasets have been collected under observance of the Declaration of Helsinki in its latest revision (2008). The retrospective epidemiological research on anonymous patient data at the University of Cologne is permitted by state law in North Rhine-Westfalia (Berufsordnung für die nordrheinischen Ärztinnen und Ärzte, https://recht.nrw.de/lmi/owa/br_text_anzeigen?v_id=70620170406111840686, last accessed August 29, 2022). The WSI dataset with class designations will be provided upon request to the authors and the Ethical Review Board of Cologne University Hospital.

## Supplemental Material

Supplemental Methods

Supplemental Results

Supplemental Table 1. Breakdown of PICASO’s performance in detecting active crescent (C-lesion) in the experiment with no external validation. In the experiment without external validation, images from all three institutes were used for training and validation. The validation performance for each institute’s dataset is reported separately. PICASO demonstrated consistent performance across datasets from all three institutes.

Supplemental Figure 1. Clustering performance of (A) PICASO and (B) Set Transformer for four synthetic test datasets. The mean of the generated mixture of Gaussians are intentionally shifted by −10 points (*i.e*., [−14, −6]), thus ensuring that data points are outside the training range. The capability of PICASO to update histopathologic vectors (HiVe) based on the input sets contributes to its superior generalization compared with the Set Transformer.

Supplemental Figure 2. Learning curves for PICASO. Training and validation loss over time is plotted for each experiment. (A) IgA nephropathy C-lesion (active crescent) detection without external validation. (B) IgA nephropathy C-lesion (active crescent) detection with external validation. (C) Case-level classification of antibody-mediated rejection (AMR).

Supplemental Figure 3. Confusion matrix for case-level classification of antibody-mediated rejection (AMR). Label 0 corresponds to non-AMR cases, and label 1 corresponds to cases with active, chronic active, and chronic AMR.
